# A Diagnostic Challenge in Linear Dermatoses: Differentiating Inflammatory Linear Verrucous Epidermal Nevus (ILVEN) Using Dermoscopy and Reflectance Confocal Microscopy—A Case Report

**DOI:** 10.3390/reports9040357

**Published:** 2026-10-16

**Authors:** Antonio Alma, Samantha Gherbassi, Alessandro Clementi, Silvana Ciardo, Francesca Farnetani

**Affiliations:** 1 Dermatology Unit, Department of Surgical, Medical, Dental and Morphological Sciences Related to Transplant, Oncology and Regenerative Medicine, University of Modena and Reggio Emilia, 41124 Modena, Italy; 2 PhD Course in Clinical and Experimental Medicine, University of Modena and Reggio Emilia, 41124 Modena, Italy

**Keywords:** inflammatory linear verrucous epidermal nevus, ILVEN, linear dermatoses, dermoscopy, reflectance confocal microscopy, case report

## Abstract

**Background and Clinical Significance**: Inflammatory linear verrucous epidermal nevus (ILVEN) is a non-melanocytic skin lesion, typically present in early childhood, that is characterized by a linear verrucous appearance distributed along Blaschko’s lines. However, cases with onset in adolescence can present a diagnostic challenge, requiring accurate distinction from other linear dermatoses. **Case Presentation**: We present the case of a 16-year-old boy with a linear verrucous lesion on his thigh showing atypical clinical features. The lesion was evaluated using dermoscopy and reflectance confocal microscopy (RCM), which revealed specific diagnostic patterns consistent with the final diagnosis. **Conclusions**: The combined use of dermoscopy and RCM provided valuable non-invasive diagnostic information, avoiding the need for an invasive biopsy, particularly in pediatric and adolescent patients.

## 1. Introduction and Clinical Significance

Inflammatory linear verrucous epidermal nevus (ILVEN) is a non-melanocytic nevus characterized by a linear and verrucous appearance and is typically distributed along Blaschko’s lines [1]. It is a rare variant of epidermal nevi, a group of hamartomatous skin lesions dominated by keratinocytic (or linear) epidermal nevi. They are thought to stem from somatic mutations resulting in genetic mosaicism, although their underlying pathophysiology is still unclear [2]. ILVEN was originally described by Altman and Mehregan in 1971 and was subsequently further characterized by Morag and Metzker [3,4].

Although it generally presents at birth or in early childhood, unconventional manifestations during or after adolescence can occur and may pose a diagnostic challenge [5].

In these cases, differentiating ILVEN from other linear dermatoses, such as linear psoriasis, lichen striatus, verrucous epidermal nevus, and porokeratosis, is essential for appropriate management, as misdiagnosis can lead to ineffective or improper treatments [1].

Although ILVEN is generally considered benign, malignant transformation has rarely been reported, including squamous cell carcinoma and basal cell carcinoma [5].

Histopathological examination represents the gold standard for diagnosis [5]. However, it can sometimes yield ambiguous or overlapping findings [6,7,8,9,10]. In such situations, non-invasive imaging techniques can provide complementary diagnostic support [1,11]. The characteristic histopathological pattern of ILVEN consists of psoriasiform epidermal hyperplasia, with sharply alternating areas of orthokeratosis with hypergranulosis and parakeratosis with a reduced or absent granular layer [12,13].

More broadly, dermoscopy, initially developed for the assessment of melanocytic lesions, has become a useful non-invasive tool in general dermatology and inflammatory dermatoses [14,15]. Similarly, reflectance confocal microscopy (RCM) has increasingly been investigated in inflammatory skin diseases, providing in vivo microscopic information that is complementary to clinical and dermoscopic examination [16,17].

Dermoscopy has demonstrated utility in clarifying equivocal clinical cases [11], showing irregular dotted vessels on a yellow-to-brown background with a verrucous papular appearance. RCM findings in ILVEN have also been reported [1]. In addition, De Luca et al. previously described both dermoscopic and confocal microscopy features of widespread ILVEN in conjunction with histopathological examinations [14].

## 2. Case Presentation

Here, we report a case of a 16-year-old male (Fitzpatrick skin type III) presenting with a linear verrucous lesion on the left upper thigh that had slowly enlarged over approximately two years. The patient was otherwise healthy, and no family history of psoriasis or epidermal nevi was reported.

The patient reported relapsing–remitting inflammation and scaly erythema.

**Figure 1:**
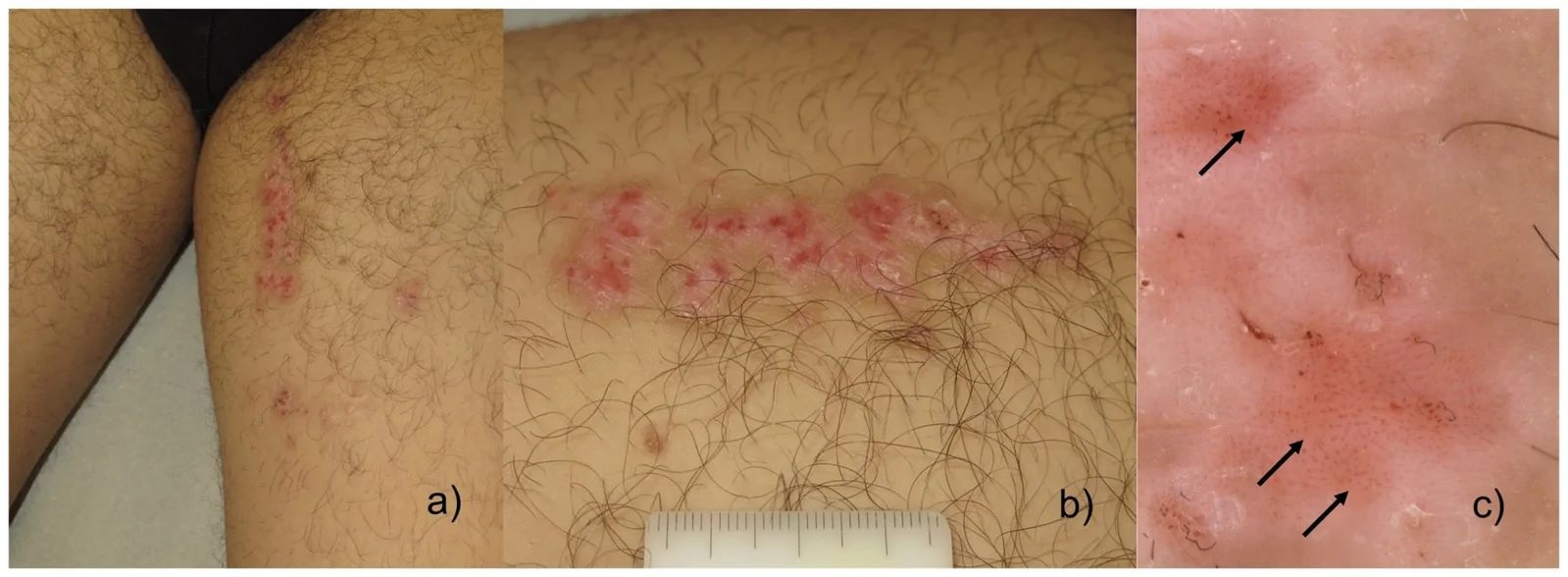
Linear verrucous lesion on the left upper thigh (**a**). Clinical examination revealed a dyskeratotic erythematous plaque with a pigmented halo and fine superficial desquamation (**b**). Dermoscopy revealed a yellow-to-brown background, moderate scales, and a dotted vascular pattern (black arrows) (**c**).

Clinical examination revealed a dyskeratotic erythematous plaque with a pigmented halo and fine superficial desquamation, features that were not pathognomonic (Figure 1). The atypical age of onset further complicated the clinical picture.

**Figure 2:**
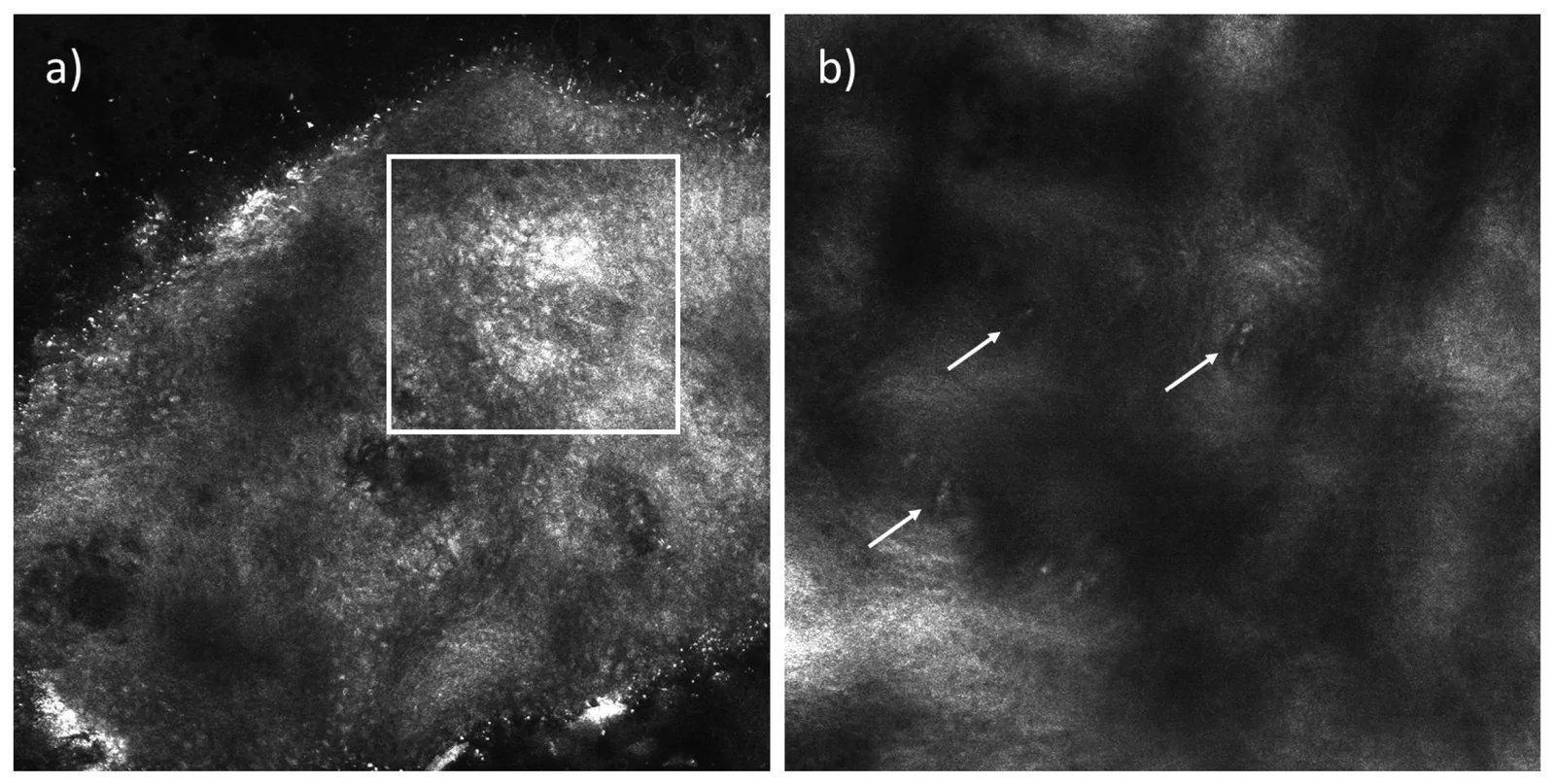
RCM showed thickened epidermal structures with hyperkeratosis and disarrangement of the keratinocytes (square) (**a**). No inflammation was detected in the superficial dermis; capillaries (arrows) were observed within the dermal papillae (**b**).

Dermoscopy (10× polarization) revealed a yellow-to-brown background with moderate scales and a dotted vascular pattern (Figure 1), while in vivo RCM (both handheld Vivascope 3000 and fixed-probe Vivascope 1500- MAVIG GmbH- were used) showed thickened epidermal structures with hyperkeratosis and disarrangement of the keratinocytes. While the epidermal findings were consistent with the previously reported RCM features of ILVEN [1], our case lacked signs of interface dermatitis and showed no inflammation in the superficial dermis (Figure 2). Dermoscopic dotted vessels corresponded to intrapapillary capillaries on RCM, consistent with a localized vascular response.

Integration of the clinical, dermoscopic, and RCM findings supported a clinical diagnosis of ILVEN; however, histopathological confirmation was not obtained.

## 3. Discussion

Linear psoriasis and lichen striatus have a similar clinical presentation and represent two principal differential diagnoses of ILVEN [10,11]. Furthermore, ILVEN should be distinguished from verrucous epidermal nevus and porokeratosis of Mibelli or disseminated superficial actinic porokeratosis [15].

Dermoscopically, linear psoriasis is characterized by a bright red background, mosaic-like vessels, and prominent scaling [16], whereas lichen striatus typically lacks marked vascular patterns and presents gray granular pigmentation and a white scar-like line with mild scales [11]. Dermoscopy of verrucous epidermal nevus typically reveals an irregular cobblestone-like surface pattern with varying shades of brown; the dermoscopic hallmark of porokeratosis is the keratin rim [17].

Using RCM, psoriasis exhibits elongated dermal papillae, dilated capillaries, and dermal inflammation [16], while lichen striatus typically presents with epidermal lichenoid infiltrates [18]—features that were absent in our patient’s lesion. RCM of verrucous epidermal nevus typically shows an epidermal honeycomb pattern and well-defined monomorphous cells demarcating each dark dermal papilla [19]; the keratin rim of porokeratosis is visible via RCM as a well-defined hyperrefractile border at the level of the corneal layer [20].

In this patient, linear psoriasis was considered less likely because the dermoscopic background was yellow-to-brown rather than bright red, and the typical mosaic-like vascular pattern was not observed [16]. Lichen striatus was less favored because the lesion showed a dotted vascular pattern rather than the gray granular pigmentation and white scar-like line typically described [11]. Verrucous epidermal nevus was considered less likely because the characteristic cobblestone-like surface pattern was not evident, while porokeratosis was not supported by the absence of a peripheral keratin rim [17].

Individually, dermoscopic findings were suggestive but not specific, as similar vascular and scaling patterns may be observed in linear psoriasis. Likewise, RCM features, although consistent with a keratinocytic disorder, were not entirely distinctive. Their combined interpretation provided concordant morphological and vascular information supporting the clinical suspicion of ILVEN.

The integration of dermoscopy and RCM provided complementary non-invasive information that strengthened the clinical suspicion of ILVEN. These techniques should not be considered substitutes for histopathology, which remains the diagnostic gold standard, but may assist clinical assessment and biopsy-site selection when histological examination is indicated.

The main limitations of this report include the absence of histopathological confirmation, the lack of documented long-term follow-up and treatment-response data, and the intrinsic depth limitation of RCM, which currently reaches approximately 230 μm and may limit evaluation of deeper structures, particularly in hyperkeratotic lesions [1]. Therefore, the diagnosis in this case should be regarded as clinically supported by dermoscopy and RCM rather than histologically confirmed.

## 4. Conclusions

The presence of a yellow-to-brown background with moderate scaling and dotted vessels on dermoscopy, together with epidermal thickening, hyperkeratosis, and keratinocyte disarray on RCM, supported the clinical suspicion of ILVEN in this atypical case. In atypical cases, the combined use of dermoscopy and RCM can provide reliable, non-invasive diagnostic support for ILVEN, reducing the need for biopsy and enabling prompt and accurate clinical management. Further studies should validate these criteria in larger cohorts.

## Data Availability

The data presented in this study are available on request from the corresponding author. The data are not publicly available due to privacy restrictions.
